# Fundamental issues in epistemic injustice in healthcare

**DOI:** 10.1007/s11019-025-10259-6

**Published:** 2025-03-07

**Authors:** Kasper Møller Nielsen, Julie Nordgaard, Mads Gram Henriksen

**Affiliations:** 1 Center for Subjectivity Research, University of Copenhagen, Karen Blixens Plads 8, 2300 Copenhagen, Denmark; 2 Psychiatry East, Region Zealand, Smedegade 16, 4000 Roskilde, Denmark; 3 Department of Clinical Medicine, University of Copenhagen, Blegdamsvej 9, 2100 Copenhagen, Denmark

**Keywords:** Testimonial injustice, Identity prejudice, Fricker, Medicine, Psychiatry

## Abstract

The research field of epistemic justice in healthcare has gained traction in the last decade. However, the importation of Miranda Fricker’s original philosophical framework to medicine raises several interrelated issues that have largely escaped attention. Instead of pushing forward, crafting new concepts or exploring other medical conditions, we suggest that it is time to take stock, reconsider, and articulate some fundamental issues that confront the field of epistemic injustice in healthcare. This paper articulates such fundamental issues, which we divide into scientific, conceptual, and theoretical issues. Scientifically, the research field is confronted by a lack of empirical evidence. It relies on cases, making generalizations impossible and the field vulnerable to bias. Conceptually, many of the claims advanced in the literature are presented as facts but are merely hypotheses to be tested. Moreover, a criterion for applying the concept of testimonial injustice in medicine is lacking, impeding the development of a construct to empirically measure said injustices. Theoretically, many of the cases discussed in the literature do not *prima facie* qualify as cases of testimonial injustice, since they lack necessary components of testimonial injustice in Fricker’s framework, i.e., being unintentional and caused by identity prejudices in the hearers. If epistemic injustice is as pervasive as it is claimed in this literature, it should be of concern to us all. Addressing the issues raised here may strengthen the conceptualization of epistemic injustice in healthcare and lead to development of constructs that finally can explore its empirical basis.

## Introduction

The research field of epistemic justice in healthcare has gained traction in the last decade (Kidd et al. 2022). The concept of epistemic injustice was coined by philosopher Miranda Fricker (2007, p. 1), who defined it as “a wrong done to someone specifically in their capacity as a knower”. Fricker (2007, pp. 28, 154, 155) originally distinguished between two kinds of epistemic injustice: (i) *testimonial injustice* in which a person “receives a credibility deficit owing to identity prejudice in the hearer” and (ii) *hermeneutical injustice* where a lacuna of collective understanding of experiences is sustained “*owing to a structural identity prejudice in the collective hermeneutical resource*”. Fricker’s concept of epistemic injustice found its way to philosophy of medicine and later philosophy of psychiatry through the publications of Havi Carel, Ian James Kidd, and Paul Crichton (Carel and Kidd 2014; Crichton et al. 2017; Kidd and Carel 2017). In healthcare, *testimonial injustice* is said to occur when patient testimonies are not given adequate credibility by clinicians due to prejudices on their part, and *hermeneutical injustice* is said to occur when patient experiences are considered unintelligible due to a lacuna in collective interpretative resources sustained by prejudices (Carel and Kidd 2014). The central claim is that patients in general and patients with mental disorders in particular suffer from a variety of epistemic injustices inflicted by clinicians and institutions (Carel and Kidd 2014; Crichton et al. 2017; Kidd and Carel 2017).

The research field has stressed the importance of sincerely listening to and considering patients’ first-person experiences of somatic illness or mental disorder, put spotlight on negative stereotypes in healthcare and on the uneven power distribution in patient-doctor interactions, and advocated substantial changes to healthcare. A recent systemic review (Côté, 2024) explored different avenues for achieving epistemic justice in healthcare. Another overview (Kidd et al. 2022) outlined developments in theoretical epistemic injustice studies as well as some of their applications to the study of epistemic injustice in psychiatry. The overview reported that the primary research focus has so far been on identifying types of epistemic injustice primarily in patient-doctor interactions and only peripherally on identifying potentially problematic structures of healthcare institutions that may produce or sustain said injustices. This overview also proposed that since the analyses in theoretical epistemic injustice studies were not developed with an eye for psychiatry, a task for the research field of epistemic injustice in psychiatry could be to adapt or create “new concepts unique to psychiatry” (Kidd et al. 2022, p. 8). The authors concluded that “much remains to be done in the conceptualization of these injustices and the ways they are generated and sustained by psychiatric practices, social and cultural conditions, and by the disruptive realities of the psychiatric conditions themselves” (Kidd et al. 2022, p. 22).

We agree with the authors of the overview that there is much to be done regarding the conceptualization of epistemic injustice in psychiatry, but we also believe that the same applies to epistemic injustice in somatic medicine. Thus, instead of pushing forward and crafting new concepts specifically for psychiatry, we believe it is time to take stock, reconsider, and articulate some fundamental issues that confront the broader research field of epistemic injustice in healthcare. The purpose of this paper is to articulate these issues. Our principal claim is that research on epistemic injustice is confronted by a palette of scientific, conceptual, and theoretical issues, all of which exert major limitations on the research field and collectively call into question if epistemic injustice is at all as widespread and pervasive a feature of healthcare as it is claimed to be in this research field.

In this paper, we primarily focus on fundamental issues related to testimonial injustice as a *transactional* injustice, i.e., injustices in interactions between patient and doctor, because testimonial injustice constituted the bulk of Fricker’s original work and because it is the primary form of epistemic injustice debated in the literature, especially in psychiatry. Although hermeneutical injustice as a *structural* concept, i.e., injustices arising due to lacunas in collective interpretative resources (Fricker 2007, p. 155), concerns other discussions and is not our primary concern here, we will nonetheless touch upon the issues of marginalization and silencing as they often intersect with testimonial injustice in this literature (cf. Dotson 2012).

The paper is structured around a discussion of specific scientific, conceptual, and theoretical issues. These fundamental issues are entangled in each other and, as we unravel different layers of their entanglement, some repetition is unavoidable, which, however, also testifies to the complexity of these issues.

## Scientific issues

Despite an increasing amount of literature on epistemic injustice in healthcare, the field is confronted by a compelling lack of empirical evidence. While Kidd et al. (2023, p. 1) claim that “there is an enormous literature testifying to negative epistemic experiences that are often interpretable as epistemic injustices”, we have not been able to identify any empirical study that has examined the magnitude of epistemic injustice in psychiatric or somatic settings. This is consistent with Carel’s (2023, italics added) statement about the newly launched ‘EPIC: Epistemic injustice in healthcare’ research project: “EPIC is the *first* research project to look systematically at epistemic injustice across several domains within healthcare and *to document empirical evidence of it*”. So far, the research field has relied on various *cases* to illustrate its arguments (see, e.g., Carel and Kidd 2014; Crichton et al. 2017; Kidd and Carel 2017; Kurs and Grinshpoon 2018; Lakeman 2010; Sanati and Kyratsous 2015; Scrutton 2017). This is perhaps an extension from Fricker’s (2007) original endeavor in the intersection between epistemology and ethics, where she primarily relied on literature and autobiographies. Case studies can have a high resolution of details, be informative, and they may be useful for teasing out intuitions and developing concepts (Sackett 1989). Although case studies can be useful, we strongly encourage the field to develop modalities of qualitative and quantitative research to document the extent and impact of the alleged epistemic injustice in healthcare. A major issue with the present literature on epistemic injustice in healthcare is that many of the claims put forth are of a general kind (see Sect. “Conceptual issues”), while relying only on case studies and theoretical conjectures (cf. Kious et al. 2023, p. 3). However, if such general claims are to be empirical tested and potentially corroborated, large-scale empirical studies using quantitative methodology are necessary. The absence of empirical studies examining the reality of these claims exerts major scientific limitations on the research field.

Another issue with the use of case studies is that they are highly vulnerable to bias, e.g., being tainted by a researcher’s own opinion. In fact, reading many of the cases in the literature on epistemic injustice in healthcare (e.g., Carel and Kidd 2014; Crichton et al. 2017) often leaves one wondering if these cases necessarily exemplify cases of epistemic injustice. Take the case that opened the research field of epistemic injustice in healthcare, namely the scene described by Carel after having shadowed a consultant pediatrician at a hospital (Carel and Kidd 2014, p. 529). A woman has just given birth, and a doctor is stitching the damages from the birth. The woman says “that really, really hurts” but nobody responds. She calls out again, this time also asking if the doctor is using anesthetics. The doctor calmly replies “there is no need. I’m nearly finished”. For Carel and Kidd (2014, p. 529), it “is hard to imagine another situation in which we would not offer pain relief to someone having a needle pushed through their genitals”, and they conclude that the woman’s testimony is “not acted upon” and her pain is “not fully registered or not considered worthy of response”. Is it a case of testimonial injustice? Maybe. It could also be the case that the doctor did in fact register and consider her complaint. If he only lacked one stitch, being “nearly finished” as he said, he would have inflicted more pain onto her, if he now were to apply a local anesthetic, which is in itself painful and which would require at least two pinpricks before the final stitch. If this was the case, then the doctor is perhaps guilty of failing to explain his decision-making, but he can perhaps not be said to be guilty of epistemic injustice.

Kenneth Kendler (2024, p. 175) has recently expressed concerns about contributions from philosophy of psychiatry, stating that “the field of mental health attracts a wide variety of theorists, some of whom are little constrained by the problems of empirical evidence”. Although Kendler’s criticism does not specifically target the research field of epistemic injustice in healthcare, it could perhaps also apply here. If the research field of epistemic injustice in healthcare is to dodge this criticism, the field needs to be mindful of its methodologies and their inherent limitations. We welcome the infusion of theories outside of medicine that may be useful for opening new perspectives or addressing questions that may have been neglected or underexplored within healthcare. However, such interdisciplinary work should be grounded in clinical reality, attentive to empirical evidence, and have, as a guiding principle, improved care of patients. If detached from the clinical reality or inattentive to evidence, there is a danger that such research in medical humanities becomes sterile and without clinical relevance or impact. In this regard, the field of epistemic injustice in healthcare must move beyond cases and launch qualitative and quantitative studies. Before empirical studies can be conducted, a method for measuring epistemic injustice in healthcare must be developed and validated, documenting that it adequately measures the specific construct it is said to measure and no other constructs (Blacker and Endicott 2008; Cronbach and Meehl 1955). The need for developing such a measure has been noted several times in the field, including in Carel and Kidd’s (2014, p. 539) seminal publication, but no measure has yet been developed.

## Conceptual issues

Given the lack of empirical evidence, one might expect that the claims made in the literature on epistemic injustice in healthcare would be tempered accordingly. This, however, is not the case. The research field is replete with strong and general claims about epistemic injustices that are not supported by empirical evidence. Most importantly, these unsupported claims are not just found randomly in the margins of the research field but even seem to constitute the field’s conceptual core, akin to a Lakatosian *hard core* (Lakatos 1970). In Table 1, we have listed a selection of such core claims in the field. Some of these claims concern the commonality, persistency, and frequency of epistemic injustice in healthcare. We also find claims about patients often being “epistemically marginalized” (Miller Tate, 2019, p. 98) and “victims of strategies of exclusion” (Kidd and Carel 2017, p. 185), where the entirety or parts of their testimony may be “excluded” or “assigned a deflated epistemic status”, especially if it is not framed in medical language (Carel and Kidd 2014, p. 530). Other claims concern negative prejudices that clinicians are said to harbor toward patients, leading clinicians to ignore, dismiss, or exclude patient testimonies. In somatic medicine, these prejudices are said to concern clinicians perceiving patients as “moaners” or “drama queens” (Crichton et al. 2017, p. 66). In psychiatry, clinicians are assumed to see patients as “cognitively impaired”, “emotionally compromised”, “existentially unstable”, or “dominated by their illness and unable to reflect on other issues” (Crichton et al. 2017, p. 66; Kidd and Carel 2018, p. 217). Claims also specifically concern clinicians’ views of patients with psychotic disorders as “unintelligible” (Miller Tate, 2019, p. 99) or “bizarre, incomprehensible, and irrational”, and then generalizing this irrationality as a feature of the patients’ “general psychic life” (Sanati and Kyratsous 2015, p. 339). In other words, clinicians are assumed to harbor the belief that psychotic patients are “*altogether* irrational and unable to make true assertions *at all*” (Carel and Kidd 2014, p. 537, italics added). Rather than picking out features of reality, the claims about clinicians harboring negative stereotypical prejudices toward patients seem to create what we, borrowing a term from John Sadler (2004), will call a “straw clinician”, i.e., a distorted image of the clinician.

**Table 1:** Core claims of epistemic injustice in healthcare

No	Claim	Example	Similar claim in other publications
1	Epistemic injustice is common in healthcare	“Epistemic injustice is a common, possibly pervasive, feature of healthcare […] it is much more likely to be systematic and extensive, rather than local and minor” Carel and Kidd (2014), pp. 538, 539	Bueter (2019), p. 1072; Drożdżowicz (2021), p. 1; Kidd and Carel (2017), p. 173; (2018), p. 211
2	Patients are often victims of strategies of exclusion	“Ill persons can be, and often are, victims of strategies of exclusion” Kidd and Carel (2017), p. 185	Carel and Kidd (2014), p. 531; (2017), p. 342; Kidd and Carel (2018), pp. 222–223; Miller Tate (2019), p. 98; Scrutton (2017), p. 349
3	Patients are often seen as objects rather than participants	“Ill persons are vulnerable to participatory prejudices [because] they are typically regarded as the *objects* of, rather than as *participants* in, the epistemic practices of medicine” Carel and Kidd (2017), p. 340	Crichton et al. (2017), p. 67; Kidd and Carel (2017), p. 179; Kurs and Grinshpoon (2018), p. 342; Scrutton (2017), p. 349; Spencer and Broome (2023), p. 15
4	Patients with mental disorders are more susceptible to epistemic injustice than patients with somatic conditions	“People with mental disorders are even more vulnerable to epistemic injustice than those with somatic illnesses” Crichton et al. (2017), p. 65	Grim (2023), p. 5876; Kidd et al. (2022), p. 7; Kurs and Grinshpoon (2018), p. 338; Miller Tate (2019), p. 97; Ritunnano (2022), p. 5
5	Patient testimonies are often dismissed due to various negative stereotypes of patients harbored by clinicians	“Testimonies of patients are often presumed to be irrelevant, unreliable, confused or otherwise lacking in credibility, owing to negative stereotypes associated with ill persons. Such stereotypes include viewing ill persons as cognitively impaired or emotionally compromised […] or as existentially unstable […] or as psychologically dominated by their illness in a way that warps their capacity to accurately describe and report their experiences (e.g., ‘the moaner’ or ‘the drama queen’ stereotype)” Crichton et al. (2017), p. 66	Carel and Kidd (2014), pp. 530, 531; Kidd and Carel (2017), pp. 177, 178; Kidd et al. (2023), p. 3; Kurs and Grinshpoon (2018), p. 339; Sanati and Kyratsous (2015), p. 483; Scrutton (2017), pp. 348, 349
6	Patient testimonies not framed in medical language are given a deflated epistemic status by clinicians	“Even if the patient’s testimony were relevant, emotionally balanced and so on, what they say is not expressed in the accepted language of medical discourse and will therefore be assigned a deflated epistemic status” Carel and Kidd (2014), p. 530	Carel and Kidd (2014), p. 535; (2017), p. 341; Freeman and Stewart (2018), p. 417; Kidd and Carel (2017), p. 179; (2018), p. 223; Scrutton (2017), p. 353
7	Clinicians find testimonies of patients with mental disorder suspect due to patients’ impaired decision-making	“The testimony of people who use mental health services is considered suspect because their capacities to make decisions are taken to be diminished. In this way, their choices and even their treatment preferences might be seen to be incoherent, illogical, or lacking credibility” Kurs and Grinshpoon (2018), p. 339	Carel and Kidd (2014), p. 537; Miller Tate, (2019), p. 99; Sanati and Kyratsous (2015), p. 483; Speyer et al. (2024), p. 2
8	Clinicians sometimes regard all aspects of a patient’s being and all testimonies as symptoms of their disorder	“The goal [of epistemic justice] is not necessarily to take all testimony at face value, but to avoid confirmation bias, whereby the individual’s psychiatric identity encompasses all facets of their being, and all testimonies become a symptom of their condition” Kidd et al. (2023), p. 3	Carel and Kidd (2014), p. 537; Scrutton (2017), p. 349; Watts (2024), pp. 2, 4
9	Patients often experience willful hermeneutical ignorance	“The resources required for the understanding of the social experiences of ill persons are not accepted as part of the dominant hermeneutical resources. Most ill persons are capable of describing their experiences in non-expert terms, but such experiences are: a. largely considered inappropriate for public discussion and b. play little or no role in clinical decision-making” Kidd and Carel (2017), p. 184	Kidd and Carel (2018), p. 220; Kurs and Grinshpoon (2018), p. 340; Miller Tate (2019), p. 98; Ritunnano (2022), p. 13; Spencer and Kidd (2023), pp. 112, 113

It bears reiteration that none of these core claims are supported by empirical evidence. From a scientific perspective, these claims are therefore best regarded as hypotheses to be tested. In the literature, however, the truth of these claims sometimes appears to be almost self-evident. For example, when later authors claim that epistemic injustice in healthcare is common and that patients with mental disorders are especially vulnerable to epistemic injustice (e.g., Bueter 2019, p. 1072; Drożdżowicz 2021, p. 1; Kidd et al. 2022, p. 7; Kurs and Grinshpoon 2018, p. 338), the authors often simply refer to the original studies by Carel, Kidd, and Crichton (Carel and Kidd 2014; Crichton et al. 2017), where these claims were first made. Whereas the original authors explicitly acknowledged the absence of empirical support, the awareness hereof seems to have dwindled in later research, where claims are simply presented with references to prior research, as if that would somehow empirically validate the claims made, which it, of course, does not.

Another conceptual issue that emerges from the case studies gravitates around the issue of *applying* epistemic injustice in healthcare. How do we determine that a patient has been subject to epistemic injustice? A look at some of the different types of cases that are referred to in the literature may perhaps provide some answers. Many of these cases are described from a third-person perspective, leaving out the patients’ first-person perspectives on situations in which they are said to be epistemically harmed (e.g., Carel and Kidd 2014; Crichton et al. 2017; Cullinan et al. 2024; Scrutton 2017). This was, for instance, the situation in the above-described case of the woman being stitched after childbirth. Was she asked if *she* experienced that her testimony had not been given uptake by the doctor? Was the doctor asked why he replied as he did? Could their answers potentially have altered the conclusion that the woman was epistemically harmed by the doctor? From this and many similar cases, we are forced to assume that first-person perspectives on said injustices are not necessary for determining that we are dealing with cases of epistemic injustice. Put differently, here, a third-person perspective seems to be sufficient for determining the presence of epistemic injustice.

Other cases in the literature, however, paint a different picture. Here, third-person perspectives are absent, and the cases consist only of patients’ first-person complaints, often centering on feelings of not being adequately listened to and reports of withholding information (so-called self-censoring) (e.g., Carel and Kidd 2014; Kidd and Carel 2017; Lakeman 2010; Scrutton 2017). From such cases, we are, by contrast, forced to assume that a first-person perspective on a given situation is sufficient for determining that epistemic injustice has occurred. Collectively, neither a first-person nor a third-person perspective is necessary for ascertaining the presence of epistemic injustice, but each perspective in itself is sufficient for making this assessment. Obviously, there is a potential tension here—what happens when a first-person and a third-person perspective on the same situation do not align? Does one perspective take priority? Or is it rather the case that if one of these perspectives sanctions epistemic injustice, then that perspective trumps the other perspective? Ultimately, it boils down to a basic question, which to the best of our knowledge has escaped attention in the research field: What is the *criterion* for determining the presence or absence of epistemic injustice in healthcare? This question stretches back to the issue concerning the absence of a method to empirically measure epistemic injustice. Such a measure can probably only be constructed once the criterion for determining epistemic injustice has been settled conceptually.

## Theoretical issues

Although the issues raised above are problematic in and of themselves, there also exist issues of a theoretical kind, stemming from the application of Fricker’s philosophical work to the domain of healthcare. Since Fricker introduced the concept of epistemic injustice, a lot of conceptual work has been carried out, and many new varieties of epistemic injustices have been suggested, for instance, Kristie Dotson’s (2011) *testimonial smothering*, Gaile Pohlhaus Jr.’s (2012) *willful hermeneutical ignorance*, Emmalon Davis’ (2018) *epistemic appropriation*, and Fricker’s own (2020) *inferential inertia* (see Kidd et al. 2017, 2022 for overviews). As stated in the introduction, we focus on Fricker’s original work, especially her account of testimonial injustice as a transactional form of injustice, since it constitutes the core theoretical framework in the literature on epistemic injustice in healthcare, and also because a closer reading of Fricker’s work may prove helpful in addressing some of the theoretical issues that permeate the research field.

On Fricker’s (2007, p. 28) original definition, testimonial injustice occurs *if and only if* a credibility deficit arises “owing to identity prejudice in the hearer”, i.e., if the hearer entertains a certain *identity prejudice* that produces this credibility deficit in the speaker. Fricker (2007, p. 27) was primarily concerned with *systematic* testimonial injustices, which involve what she called “tracker prejudice”, i.e., “those prejudices that ‘track’ the subject through different dimensions of social activity”, and which leads to “a gamut of different injustices”, and which is thus of concern to *social justice*. By contrast, Fricker (2007, pp. 28, 29) was not primarily concerned with *incidental* or *localized* cases of testimonial justice, even though they might have unfavorable consequences—e.g., being prejudiced by scientists at a science conference based on the identity prejudice of being a philosopher of science. The crucial component to notice in these gradations of testimonial injustice is that they must derive from what Fricker (2007, pp. 22, 60) called the “ethical poison” of prejudice, i.e., speakers in epistemic undertakings such as testimonies, assertions, telling, questions, etc., receive a credibility deficit based on an identity prejudice in the hearer. Cases of innocent error or “epistemic bad luck”, even though they might hinder epistemic exchange, should, on her account, not be counted as cases of testimonial injustice if they do not involve the “ethical poison” of identity prejudice (Fricker 2007, pp. 21, 22, 41–43).

From Fricker’s original work, we can now see that also theoretical problems crop up with many of the cases discussed in the literature on epistemic injustice in healthcare. First, often we cannot tell—given the brevity and decontextualized nature of the cases—whether they involve any *identity prejudice* at all. If that cannot be satisfactorily established, we cannot conclude that the cases concern testimonial injustice. Consider one last time the case of the woman being stitched after childbirth. What identity prejudice is the doctor supposed to be entertaining? A similar question applies to the three cases described by Crichton et al. (2017, pp. 66, 67) as well as to many other cases (e.g., Kidd and Carel 2017; Kurs and Grinshpoon 2018; Scrutton 2017). Take the case described by Crichton et al. (2017, p. 66), where a young man was admitted to a psychiatric ward, claiming to be a relative of the then Soviet leader, which was believed to be a delusion, but turned out to be true. Such cases may simply be innocent errors, which do not *prima facie* involve anything ethically and epistemically culpable. Yet, even if we accept that there is something epistemically culpable involved in the cases, these errors are still ethically non-culpable, as long as they do not involve the “ethical poison” of identity prejudice (Fricker 2007, p. 22). As Kious et al. (2023) have pointed out, such cases are probably simply cases of inadequate clinical care—innocent errors or epistemic bad luck—that do not involve the ethical poison of identity prejudice required for qualifying as cases of testimonial injustice. To make them cases of epistemic injustice, we would have to assume that we *know* what drives the clinical decisions, namely identity prejudice. But in fact, we do not know if prejudice played any role. Finally, even if we go as far as to assume that the patients in some of these cases received a credibility deficit due to an identity prejudice in the clinician, these cases may still well be *incidental* rather than *systematic*, and there is nothing in these cases to suggest that they track the patients across different contexts.

Moreover, on Fricker’s (2017) account, identity prejudices must be *unintentional*. By contrast, clinicians may *intentionally* disregard certain parts of patient testimonies, but this will thus not constitute testimonial injustice on Fricker’s account. In both somatic medicine and psychiatry, clinicians may have good reasons for disregarding some parts of patient testimonies. This touches upon the issue of information relevancy, where some contributions in the patient-doctor dialogue often are irrelevant. We agree with the statement of the doctor quoted by Carel and Kidd (2014, p. 530), who said, ‘‘patients say a lot of irrelevant things like ‘when I eat lettuce my elbow hurts’. I have to listen carefully for the important stuff and ignore the rest”. Another example could be that of a mother calling the doctor because her child has a high fever and some red, punctiform, round spots on the chest. In the example in the quote from the doctor, the testimony of the elbow hurting when eating lettuce is medically irrelevant. In the other example, however, the testimony is highly relevant and should lead to instant medical action. Generally, doctors should be interested in hearing as many details of patient testimonies as possible, because medically important information otherwise could be missed. However, not all information provided by patients is medically relevant, and some information can safely be ignored. Of course, patients should not be blamed for providing this information—how should they know what is medically relevant? Deciding what is medically relevant and what is not requires medical expertise. Intentionally disregarding some parts of patient testimonies, because they are medically irrelevant for the examination at hand, does not amount to testimonial injustice. To make it a case of testimonial injustice, we would again have to assume that we know what drives the clinicians in disregarding parts of patient testimonies, namely, an *unintentional* identity prejudice entertained by the clinician toward the patient instead of an *intentional* relevancy assessment occasioned by medical expertise. By making this assumption we are, of course, skating on thin ice.

This issue ties back to the unsettled question of a first-person vs. third-person criterion for determining whether something is testimonial injustice in somatic medicine and psychiatry. From a Frickerian perspective, we cannot rely solely on patients’ first-person complaints of testimonial injustice, because this would make it difficult to determine whether clinicians entertained identity prejudices that were driving the credibility deficit ascribed to the patients. Testimonial injustice concerns the hearers’ psychology and their biases. By solely relying on first-person reports of being epistemically wronged and not concerning ourselves with whether identity prejudice is involved, we run the risk of making testimonial injustice overly subjective and claims thereof unfalsifiable. Thus, if the field of epistemic injustice in healthcare is to launch the much-needed empirical studies, it must move beyond the dichotomization of first-person and third-person perspectives as described above. Given Fricker’s emphasis on identity prejudice in the hearer and the unintentional nature of prejudice, we propose that the research must address both the patient’s perspective, i.e., the experiences of epistemic injustice, and the doctor’s perspective, i.e., the identity prejudices said to be enacted by clinicians. We strongly encourage the development of checklists or rating scales that can measure these experiences and biases (cf. Fava et al. 2012; Feinstein 1987). To this end, qualitative studies may be a good place to start ‘bootstrapping’ to gradually construct and later validate measures of epistemic injustice (cf. Cronbach and Meehl 1955, p. 7). We are not under the illusion that this will be an easy task. Examining identity prejudices, let alone prejudices clinicians are said to harbor toward the patients they treat, minding also that such prejudices, following Fricker, must be unintentional in nature, requires a sophisticated method. The field may want to find inspiration in other research fields, e.g., studies empirically assessing implicit bias (e.g., Greenwald et al. 2022; Maina et al. 2018).

Another issue that permeates the research field on epistemic injustice in healthcare concerns the claims of serious harms of epistemic injustice (Kidd et al. 2023, p. 3). Spencer and Kidd (2023, p. 109), for instance, claim that: “being wronged epistemically can be seriously problematic and sometimes even fatal”. Beyond *cases* of serious harm and even death that are attributed to epistemic injustice in this literature (e.g., Crichton et al. 2017; Freeman and Stewart 2019; Sanati and Kyratsous 2015), the empirical evidence supporting these claims is also absent. Fricker (2007, pp. 43–59) herself distinguished between *primary* and *secondary* harms associated with testimonial injustice, where the secondary kind splits into *practical* and *epistemic,* with the former having more immediate practical impact, such as being unrightfully fined, and the latter concerns being a provider of knowledge. According to Fricker (2007, pp. 132–134), the *primary* harm of testimonial injustice is *epistemic objectification*, where the subject is deprived of her ability to provide knowledge and thus becomes a *mere* “source of information” (see also McGlynn 2021). This demotion from a subject to a mere object deprives the subject of something foundationally human, what Pohlhaus Jr. (2014) has called “truncated subjectivity”. According to Fricker (2007, pp. 53–55, 58, 145), being an informant, a knower, plays a central role in “steadying the mind”, developing “personhood”, and injustices of this form “can cause deep and wide harm to a person’s psychology and practical life”. Fricker’s own discussion, however, is primarily philosophical with few references to empirical evidence of these harms. As Fricker (2007, p. 58) herself acknowledges, these questions of harm must be settled empirically, and her analysis should only serve as “pregnant speculation as to the ramifications in a person’s life”. One should, however, be careful with extrapolating these philosophical reflections on the potential harm of epistemic injustice into psychological constructs. We know from the field of microaggressions, which has also been related to epistemic injustice (so-called epistemic microaggressions) (Freeman and Stewart 2018, 2019), that the relationship between experienced microaggressions and detrimental mental health outcomes is not straightforward (Lilienfeld 2017, 2020).

A third issue concerns the expansion of the field of epistemic injustice with the introduction of various additional concepts and the application of this theoretical apparatus to other fields. Fricker’s central cases of testimonial injustice were distinct and involved tracker prejudices. Besides authors relying “on under-articulated accounts of epistemic injustice” (Kidd et al. 2022, p. 4), expansion of the concept has introduced fuzziness to the very concept of epistemic injustice. Fricker (2017, p. 53) herself advocates a “continued strictness” with the concept of testimonial injustice, arguing that the concept “will only be useful if it remains bounded and specific, not relaxing outwards to embrace the generality of unfair interpersonal manipulations” (see also Kidd et al. 2023, p. 1). Lauren Freeman and Heather Stewart (2018, p. 417, italics added), for instance, advance the concept of epistemic microaggressions, which they define as “*intentional or unintentional*/unconscious slights conveyed in speech or gesture by healthcare providers that dismiss, ignore, ridicule, or otherwise fail to give uptake to claims made by patients”. Such broad concepts that rely on the “experiences of victims” rather than “the intent of the perpetrator” (Freeman and Stewart 2019, p. 122) come with the danger of excessively broadening the concept of epistemic injustice, especially testimonial injustice. This may result in what Mats Alvesson and Martin Blom (2020) have called *hembigs*: *hegemonic*, *ambiguous*, and *big concepts* (cf. Haslam 2016; Solomon 2024). Not all errors or injustices in healthcare are ‘epistemic’ in nature in the sense described in our paper, yet many different cases of patient complaints utilized in this literature are simply equated with epistemic injustice (e.g., Carel and Kidd 2014, 2017; Lakeman 2010). This also relates to the assumed distinctiveness of epistemic injustice, where Kidd et al. (2022, p. 2) have argued that “epistemic injustice as a concept is distinctive; it is not simply a restatement, in a different vocabulary, of experiences and social processes already described by stigma and other concepts”. If this is true, research on epistemic injustice cannot piggyback on empirical stigma research or simply construe patient complaints in healthcare as being expressions of epistemic injustice.

Moreover, it is crucial to remember that Fricker’s (2007, pp. 22, 23; 2017) original work on epistemic injustice had a specific scope, namely, questions of social justice and the experiences of marginalized groups, such as women, Black people, the working class, and sexual and religious minorities, etc. Patients with somatic illness or mental disorders are as a group unfortunately poised to various forms of social injustice, marginalization, and discrimination (Stangl et al. 2019; Thornicroft 2006). However, unlike the groups described in Fricker’s work, who have no diminished epistemic abilities (i.e., abilities to provide testimony, pose questions, assert prior events, make value judgements, etc.), the situation can be different in healthcare. Some patients with mental disorder or somatic illness may exhibit some degree of diminished epistemic abilities, episodically or more enduring, e.g., due to psychosis, delirium, or cognitive deficits (e.g., in dementia). The role of such diminished epistemic abilities seems, however, often to be insufficiently acknowledged in the literature on epistemic injustice in healthcare.

Finally, let us cautiously remark that the importation of conceptual pairs such as victim/perpetrator, oppressed/oppressor, and marginalized/dominant from theoretical epistemic injustice studies to the domain of healthcare may not only prove barren but also counterproductive in capturing the complexities of the patient-doctor relation (e.g., Kidd and Carel 2017; Miller Tate 2019; Spencer and Kidd 2023). Framing the patient-doctor relation in terms of binary, value-laden, and antagonistic schemas seems unfruitful for capturing a complex relationship that is perhaps better understood as a *dialogical I-Thou relationship* (León et al. 2022; Stanghellini 2007). This is not to deny that oppressive or paternalistic relationships may occur but simply framing patient-doctor relations in such binary terms is something of a caricature.

For decades, medical ethics have been at the forefront promoting clinical care and patient rights (e.g., autonomy regarding treatment) as well as regulating studies involving human subjects (e.g., informed consent), with powerful associations rising to give voice to patients and engaging with healthcare providers and policy makers (Neuberger 2015). Healthcare has undeniably come a long way from what sometimes is referred to as the “doctor knows best” attitude or the so-called Hegelian principle of “what is useful is right” (Neuberger 2015). Alongside, there has been an ongoing debate of what term should be used to refer to persons receiving medical help such as ‘patients’, ‘clients’, ‘users’, or ‘consumers’, perhaps especially within mental health care, with different reasons for and against each term (e.g., Fischer et al. 2020; Torrey 2011). We stick to the traditional term ‘patients’ (also preferred by some healthcare recipients (Costa et al. 2019)), because, in the clinical encounter, a person seeks medical help for a problem from which she suffers (the meaning of ‘patient’ is ‘to suffer’). Despite the progress in healthcare outlined above, it is important to stress that the patient-doctor relation is inherently asymmetrical (which the terms ‘clients’, users’, or ‘consumers’ potentially conceal), because one part takes on the responsibility of caring for the other part. Taking on the responsibility of care does not imply imposing passivity or decreased agency on the part of the patient, and the nature of this responsibility and how it may be clinically enacted depends on the condition being treated (e.g., a heart attack, cancer, or asthma). Describing the patient-doctor relation, Edmund Pellegrino (1979) made an important remark when stating that when we become ill, we suffer an insult to “our whole being”, a restriction of our freedom, and to regain our freedom, we place ourselves “in the power of another human”. The ideal patient-doctor relation can perhaps best be described as centered on an equilibrium of paternalism and autonomy, favoring a deliberative model, where shared decision-making on appropriate treatment plays a significant role as well as recognition of the patient’s values (Emanuel and Emanuel 1992).

In achieving this equilibrium between paternalism and autonomy, listening to patient testimonies throughout the process of diagnosis and treatment is quintessential. Still, patients may experience aspects of a diagnostic assessment (e.g., a lumbar puncture) or of a treatment (e.g., chemotherapy) as unpleasant. Sometimes, the responsibility of caring for patients may even involve acting against the patients’ will (e.g., by involuntarily admitting patients who are dangerous to themselves or others). This constitutes a kind *benevolent* objectification and paternalism, which unfolds within a dialogical relationship of understanding the patient’s needs, values, and context (Christoff 2014; Svenaeus 2023). Doctors should, of course, be attentive to the asymmetrical relation, the power and responsibility that comes with it, and be mindful of one of the basic principles in medical ethics, namely, above all, do no harm (*primum non nocere*).

## Conclusion

In the last decade, epistemic injustice has become a popular concept in the domain of healthcare. However, the importation of Fricker’s philosophical work on epistemic injustice in epistemology and ethics to the domain of healthcare is not smooth sailing but rather raises a series of interrelated issues that must be addressed. If epistemic injustice in healthcare exists in the form or degree hypothesized in the literature, it should be of significant concern to us all. Consequently, this topic must be explored thoughtfully and with methodological rigor. In this paper, we have highlighted some fundamental issues in the research field. If adequately resolved, we believe that it will substantially strengthen the conceptualization of epistemic injustice in healthcare. This, in turn, is crucial for developing and validating a testable measure that finally may enable empirical assessments of epistemic injustice in healthcare. If adequate empirical evidence cannot be secured, one must be skeptical about the existence of epistemic injustice as a widespread and pervasive feature of healthcare.
